# Investigation of Radiation Exposure of Medical Staff During Lateral Fluoroscopy for Posterior Spinal Fusion Surgery

**DOI:** 10.3390/jcm13216442

**Published:** 2024-10-27

**Authors:** Seiya Watanabe, Kazuo Nakanishi, Masakatsu Mura, Ato Yutori, Go Hitomi, Kazuya Uchino, Hideaki Iba, Yoshihisa Sugimoto, Shigeru Mitani

**Affiliations:** 1 Department of Orthopaedic Surgery, Kawasaki Medical School, 577, Matsushima, Kurashiki 701-0192, Okayama, Japan; k.nakanishi@med.kawasaki-m.ac.jp (K.N.); kazuya_u@med.kawasaki-m.ac.jp (K.U.); i-hideaki@med.kawasaki-m.ac.jp (H.I.); y.sugimoto@med.kawasaki-m.ac.jp (Y.S.); ms.44100.kawasaki@gmail.com (S.M.); 2 Department of Radiology, Kawasaki Medical School, 577, Matsushima, Kurashiki 701-0192, Okayama, Japan; muramasa@med.kawasaki-m.ac.jp (M.M.); yutori@med.kawasaki-m.ac.jp (A.Y.); hitomi@med.kawasaki-m.ac.jp (G.H.)

**Keywords:** radiation exposure, aerial dose distribution map, lateral fluoroscopy, posterior spinal fusion, ICRP, intraoperative fluoroscopy

## Abstract

**Background/Objectives:** In spinal surgery, it is especially crucial to insert implants in the correct location. Intraoperative fluoroscopy is often necessary to safely perform spinal surgery because of serious complications that can occur if the screw deviates. However, the use of intraoperative fluoroscopy comes at the cost of radiation exposure to the surgeons and operating room staff. Therefore, it is desirable for spinal surgeons to understand the characteristics of radiation in order to minimize patient and medical staff exposure. This study aimed to create an aerial radiation dose distribution map for lateral fluoroscopy, a commonly used technique for posterior spinal fusion. **Methods:** A human body-equivalent phantom was placed in a prone position on the Jackson Table. The measurement method used was a lateral fluoroscopic evaluation, assuming posterior spinal fusion. Measurements were taken at three levels: 80 (gonadal), 100 (thoracoabdominal), and 150 cm (lens and thyroid). **Results:** The highest radiation doses were received by primary surgeons. The scrub nurse was the next most exposed. **Conclusions:** We developed an aerial dose distribution map for lateral fluoroscopy in posterior spinal fusion. Radiation exposure was the highest among primary surgeons.

## 1. Introduction

In spinal surgery, it is especially crucial to insert implants in the correct location. Because of the serious complications that can occur if a screw deviates [1], intraoperative fluoroscopy is often necessary to safely perform spinal surgery. Deviation of the Pedicle Screw (PS) in the thoracolumbar spine can result in spinal cord injury, the leakage of cerebrospinal fluid due to dural injury, vascular injury, inadequate fixation, and iatrogenic fractures. Zhao et al. reported that complications occurred in 46 of 781 (5.9%) patients who underwent surgery [2]. Vascular injuries were caused by screws being inserted deeper medially than appropriate, resulting in abdominal aortic injuries. The incidence of abdominal aortic injury was 0.13%. As for the spinal cord injury, it was caused by penetration of the pedicle and straying into the spinal canal. Cerebrospinal fluid leakage had a 0.4% chance of occurring. In addition, the screw was not inserted in the proper position, resulting in weak fixation and postoperative implant breakage [2].

Vertebral artery injuries in cervical spine surgery are estimated to be about 0.08%. Of these, 1.35% were due to screw deviation in C1/2 posterior fixation and 0.2% in C3-6 posterior fixation. A total of 23% of patients in whom vertebral artery injury occurred were critically life-threatening due to cerebellar or brainstem infarction [3].

In addition to intraoperative fluoroscopy, there are other techniques that can improve screw insertion accuracy. Navigation systems such as C-arm and O-arm [4] Cervical pedicle screw guide with a template [5] and robot-assisted placement of pedicle screws during spine surgery [6].

Spinal navigation systems are intended to improve screw insertion accuracy and reduce radiation exposure. Navigation systems consist of many components that must work in concert. Typically, an imaging mechanism is used to collect X-ray images, which are imported into a computer workstation to create a three-dimensional (3D) reconstruction of the anatomy of the subject [7]. This computer system interacts with specialized optical cameras and surgical tools to guide real-time screw insertion without the need for repeated fluoroscopic images [7]. When navigation was first developed, preoperative images were used, but there has been a gradual shift to the use of intraoperative 3D images [8]. Preoperative images do not represent anatomical changes due to intraoperative positioning, so intraoperative images represent more anatomical structures [9]. The 3D C-arm is a CT-based navigation that collects images from a 190° screening arc [10]. Up to 200 fluoroscopic images are collected at equidistant angles to reconstruct a 3D view of the anatomy of the spine. Medical staff are removed from the operating room during image creation, reducing unnecessary radiation exposure. In terms of radiation exposure, previous studies showed reduced radiation exposure with the use of the 3D C-arm compared to standard fluoroscopy [11]. Kim et al. performed one such study in 18 cadaveric spines undergoing minimally invasive transforaminal lumbar interbody fusion (MIS TLIF). The authors demonstrated that the fluoroscopy time was lower compared to the standard fluoroscopy group (28.7 s vs. 41.9 s) [12]. The advantage of the 3D C-arm is that multiple studies have reported better accuracy of the pedicle screw when compared to standard fluoroscopy [11,12]. The O-arm is a cone-beam, CT-based intraoperative imaging modality that can produce a 360° scanning arc. O-arm devices can acquire up to 750 images in a single scan, and these images can be utilized with navigation systems to create 3D anatomical reconstructions [13]. The O-arm is also programmed with preset modes that optimize kilovoltage and milliampere settings for various patient sizes and anatomical regions [14]. Like the C-arm, the O-arm also reduces radiation exposure by allowing medical staff to step outside the operating room during image creation. Although navigation is useful in reducing radiation exposure, it is estimated that only about 11% of spine surgeons use navigation systems [15]. Factors that hinder the use of navigation systems include increased costs and prolonged operating time [15].

The insertion accuracy of the cervical pedicle screw guide with a template is 95.3%, which is high insertion accuracy. The disadvantage, however, is that proper placement of the template requires extensive soft tissue deployment and time for template creation, making it impossible to use in emergency surgeries [4]. The advantages of robot-guided thoracolumbar screw placement include improved accuracy and safety and reduced radiation exposure to medical staff. Disadvantages include screw deviation due to mechanical malfunction, the computer system, robotic arm set-up, software start-up, etc., which lengthen the operation time compared to conventional fluoroscopy, and increase high operating costs, which limit the number of facilities that can use it [5].

The deviation rate for freehand insertion of the PS without intraoperative fluoroscopy was reported to be approximately 10.5% [16]. However, the use of intraoperative fluoroscopy comes at the cost of radiation exposure to the surgeons and operating room staff. Radiation exposure affects thyroid cancer, heart disease, cataracts, and genital organs [17,18,19,20,21]. The thyroid gland has a high metabolic activity and rapid turnover of cells, making it especially radiosensitive. Exposure to radiation is implicated in the pathogenesis of thyroid disorders, including adenomas, thyroiditis, hypothyroidism, and malignant neoplasms. Thyroid carcinoma can result from direct exposure to ionizing radiation. Up to 85% of papillary thyroid carcinomas are radiation-induced [22]. The onset of cataracts is one of the better documented sequelae of ionizing radiation exposure to the eye. The etiology of cataracts involves clouding of the normally clear lens of the eye, which may obstruct vision. Cataracts are further classified by the anatomic location of opacification: nuclear, cortical, posterior, or mixed. Opacification of the posterior lens, in comparison to the other locations, is relatively specific to radiation exposure. The association between ionizing radiation and cataract development was established from the observation of whole-body radiation for the treatment of patients with leukemia and other medical conditions, as well as the observation of atomic bomb survivors from World War II [23]. The effects of radiation on cardiovascular tissue can result in a wide range of diseases, including coronary artery disease, cardiomyopathy, conduction system abnormalities, pericardial disease, and valvular heart disease. Valvular heart disease and coronary artery disease can develop decades after radiation exposure [18]. The effects of radiation exposure on the genital organs are varied, including impaired spermatogenesis, radiation-induced urethral stricture, and radiation-induced vaginal stenosis [24,25,26]. Effects on the skin include skin ulcers, skin cancer, hair loss, skin necrosis, and epidermal necrosis caused by acute and high-dose radiation exposure. In addition, long-term low-dose radiation exposure causes finger dermatitis and fingernail pigmentation [21]. The cancer incidence rate among orthopedic surgeons is five times higher than that among physicians not exposed to radiation [27]. Furthermore, spinal surgeons are exposed to more radiation than other orthopedic specialists [28,29]. Therefore, it is desirable for spinal surgeons to understand the characteristics of radiation in order to minimize patient and medical staff exposure. This study aimed to create an aerial radiation dose distribution map for lateral fluoroscopy, a commonly used technique for posterior spinal fusion, and to determine the radiation exposure for each position of the radiation worker.

## 2. Materials and Methods

**Figure 1:**
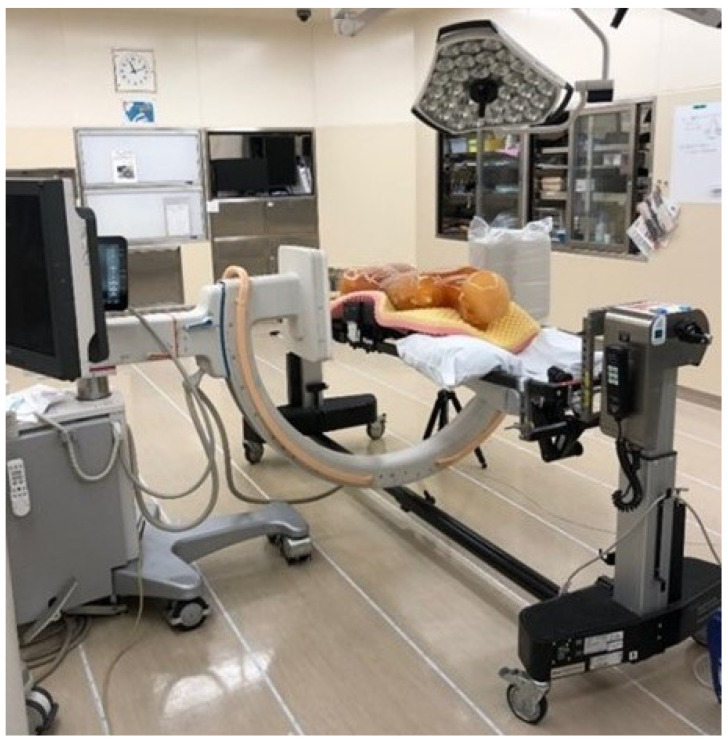
A human body-equivalent phantom is placed in the prone position on the Jackson table. The operating room is arranged in a grid with 50 cm intervals.

**Figure 2:**
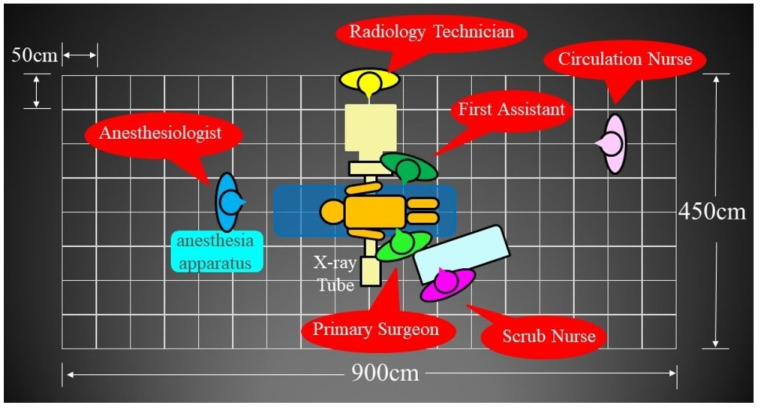
The assumed location of the medical staff.

A diagram of the experimental set-up is shown in Figure 1. A human body-equivalent phantom (CT human torsophantom CTU-41, Kyoto Scientific, Kyoto, Japan; product size: 100 cm, 45 kg) was placed in the prone position on a Jackson Table (Howell Medical, Harbin, China). The potentiometer was RAMTEC 1000D (Toyo Medic, Tokyo, Japan), the ionization chamber was RC180 (Radcal Corporation, Monrovia, CA, USA), and the fluoroscopy system was Zenition70 (PHILIPS, Amsterdam, The Netherlands). The measurement method used was a lateral fluoroscopic evaluation, assuming posterior spinal fusion. The measurement point was centered on the third lumbar vertebra. The operating room was arranged in a grid at 50 cm intervals. We placed the ionization chamber at the intersection of the grids and measured three levels: 80 cm (gonadal elevation), 100 cm (thoracoabdominal), and 150 cm (lens and thyroid elevation). Irradiation conditions were 87 kV, 2.74 mA, and 7.5 pulses/min. The irradiation time was set to 1 min. Fluoroscopy is placed in front against the third lumbar vertebra. We rotated 90° from there for lateral fluoroscopy. The object was then 55 cm from the X-ray tube and 45 cm from the detector. SS-3000 (SS Giken Co., Ltd., Aichi, Japan) was used to produce the aerial radiation dose distribution map. The assumed locations of the medical staff are shown in Figure 2. Because the radiation weighting factor of X-rays is 1, the absorption dose (Gy) and equivalent dose (Sv) are assumed to be equal [30]. Radiation doses in this study were measured in μGy/min.

## 3. Result

**Figure 3:**
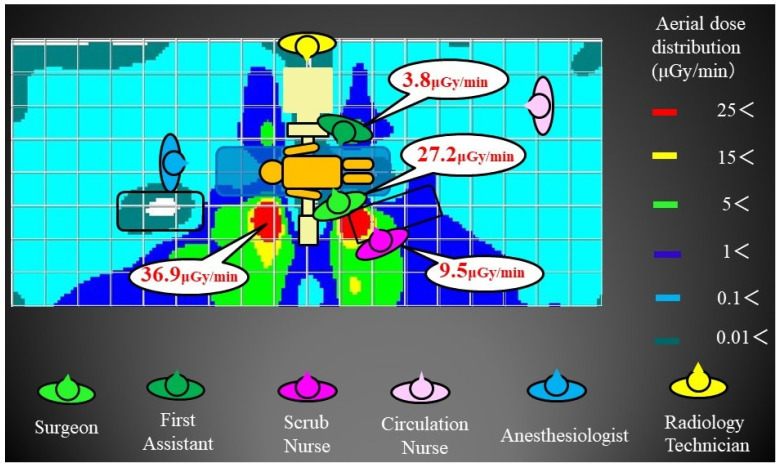
Aerial radiation dose distribution map at 80 cm: The primary surgeon was in the high-dose range of 27.2 μGy/min. The first assistant was 3.8 μGy/min, and the scrub nurse was 9.5 μGy/min. Anesthesiologists, circulation nurses, and radiology technicians were in the low-dose range.

**Figure 4:**
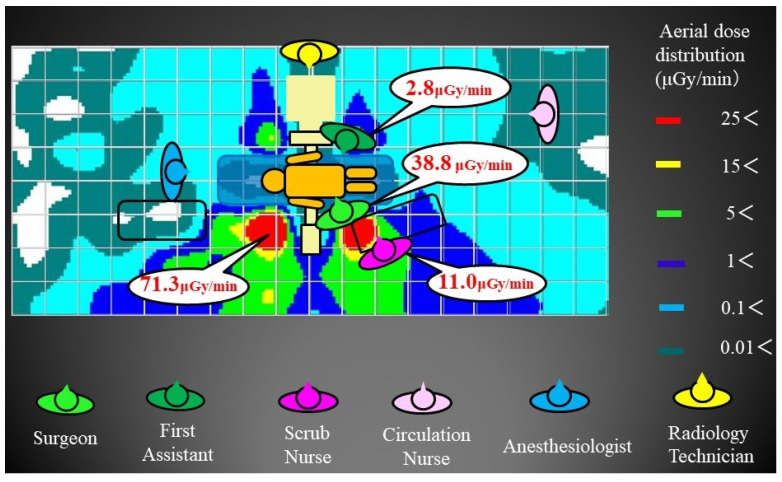
Aerial radiation dose distribution map at 100 cm: The primary surgeon was in the high-dose range of 38.8 μGy/min. The first assistant was 2.8 μGy/min, and the scrub nurse was 11.0 μGy/min. Anesthesiologists, circulation nurses, and radiology technicians were in the low-dose range.

**Figure 5:**
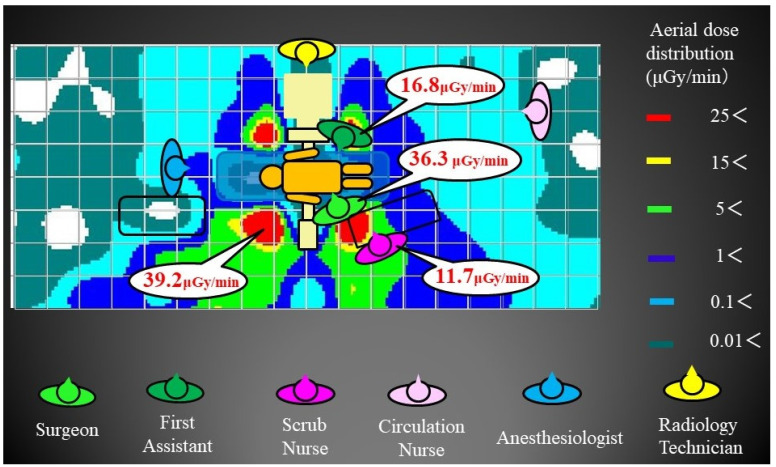
Aerial radiation dose distribution map at 150 cm: The primary surgeon was in the high-dose range of 36.3 μGy/min. The first assistant was 16.8 μGy/min, and the scrub nurse was 11.7 μGy/min. Anesthesiologists, circulation nurses, and radiology technicians were in the low-dose range.

The highest aerial dose distribution at the 80 cm (gonad) elevation was 36.9 μGy/min on the head side across the X-ray tube from the standing position of the primary surgeon. The primary surgeon was in the high-dose range of 27.2 μGy/min. The first assistant was 3.8 μGy/min, and the scrub nurse was 9.5 μGy/min. Anesthesiologists, circulation nurses, and radiology technicians were all in the low-dose range of 1 μGy/min or less (Figure 3). The highest dose range at the 100 cm (thoracoabdominal) elevation was 71.3 μGy/min on the head side across the X-ray tube from the primary surgeon’s standing position. The primary surgeon was in the high-dose range of 38.8 μGy/min. The first assistant was 2.8 μGy/min, and the scrub nurse was 11.0 μGy/min. Anesthesiologists, circulation nurses, and radiology technicians were all in the low-dose range of 1 μGy/min or less (Figure 4). Similarly, the highest dose range in the aerial radiation dose distribution map at the 150 cm (lens and thyroid) elevation was 39.2 μGy/min at the head side of the primary surgeon. The primary surgeon was in the high-dose range of 36.3 μGy/min. The first assistant was 16.8 μGy/min, and the scrub nurse was 11.7 μGy/min. Anesthesiologists, circulation nurses, and radiology technicians were all in the low-dose range of 1 μGy/min or less (Figure 5). As for the primary surgeon, the dose range was as high as 25 μGy/min at all elevations. The first assistant had doses of 5 μGy/min or less at the 80 cm and 100 cm elevations, but only at the 150 cm elevation was the high-dose range of 25 μGy/min or more, similar to that of the primary surgeon. For the scrub nurses, the dose range was 9–12 μGy/min at any height, which was the second highest after the primary surgeon. Anesthesiologists, circulation nurses, and radiology technicians were all in the low-dose range, but there were almost no areas in the operating room where the dose was 0 μGy/min.

## 4. Discussion

Radiation technology has brought revolutionary advances in diagnosis and treatment over the past 100 years. The first medical use of radiation was initiated in 1895 by German physicist Wilhelm Röntgen [31]. At the same time, Thomas Edison in 1895 greatly advanced technology using fluorescent screens and made the Edison Vitascope available for home fluoroscopy in 1896 [32]. The causes and effects of radiation exposure on carcinogenesis and injury were unclear and debated during the first three decades of the use of radiation for diagnostic imaging.

In recent years, minimally invasive spinal treatments have been required because of the super-aging society. However, minimally invasive spine surgery requires fluoroscopic or navigational confirmation because of the difficulty in anatomical orientation with the naked eye. Because navigation is expensive, facilities without it must rely on fluoroscopy. The operating room is also the site for the medical team. Surgery is performed not only by the surgeon but also by assistants, scrub nurses, circulation nurses, anesthesiologists, radiologists, and many others. Therefore, spine surgeons need to provide adequate protection against radiation exposure not only for themselves but also for their medical staff.

The International Commission on Radiological Protection (ICRP) identified justification, optimization, and dose limitation as the three principles of radiation protection. Justification and optimization are often applied to patients, and dose limits are applied to medical staff. The limits for occupational exposure, defined by the ICRP in 1990, are 150 mSv for exposure to the lens, 500 mSv for exposure to the skin, and 20 mSv for the effective dose, which indicates the dose to the whole body in one year [33]. In 2012, the equivalent dose limit for the lens was further lowered to less than 20 mSv per year, which is a significant reduction from the standard [34]. To reduce radiation exposure, it is necessary to strictly adhere to three principles of radiation protection: time, interception, and distance. In addition to strictly adhering to these three principles, proper knowledge of occupational exposure is necessary. This study aimed to clarify the exposure status of radiation workers in the operating room in their respective positions.

In this study, the surgeon’s radiation exposure was 27.2 μGy/min at the 80 cm height, 38.8 μGy/min at the 100 cm height, and 36.3 μGy/min at the 150 cm height. The average lateral fluoroscopy time for posterior spinal fusion at our hospital was approximately 4 min per case. Therefore, per case, the primary surgeon will have approximately 108.8 μGy at the 80 cm height, 155.2 μGy at the 100 cm height, and 145.2 μGy at the 150 cm height. In addition, since an average of about 150 cases of posterior fixation is performed at our hospital per year, the average is 16,320 μGy (16.32 mGy) at the 80 cm height, 23,280 μGy (23.28 mGy) at the 100 cm height, and 21,780 μGy (21.78 mGy) at the 150 cm height. A lead apron for radiation exposure at 80 cm and 100 cm elevations and lead glasses at 150 cm elevation, is used in order to reduce radiation exposure doses. Chauhan et al. reported that a lead apron of 0.35 mm can shield approximately 95% of the radiation dose [35]. In addition, in the 150 cm high lens area, radiation exposure was reduced by approximately 60% by wearing radiation-protective eyewear [36]. In this study, the estimated value for lateral fluoroscopy alone in lens spinal surgery was 21.78 mGy, which would be expected to exceed the reference value if radiation-protective glasses were not worn. However, other institutions reported that the radiation time per spinal surgery case ranged from 0.2 to 1.4 min, which was considerably shorter than the radiation time at our hospital [37]. Furthermore, Cheriachan et al. measured radiation exposure at eye level for orthopedic surgeons, which is well below the ICRP limit of 20 mSv of radiation per year, so wearing radiation protection glasses is not necessary [38]. The time required for radiation exposure is largely dependent on the skill of the primary surgeon. There is concern that residents may unconsciously irradiate themselves, resulting in longer radiation exposure times. We guess that the reason why our hospital tends to have longer radiation exposure time per case than reported by other facilities is due to the regular rotation of young residents. We believe that radiation protection glasses are necessary because the radiation exposure time may be longer than expected at our hospital. In addition, depending on the shape of the radiation protection glasses, the sides may not be shielded and exposure may occur. Therefore, when radiation is generated, the medical staff should face the fluoroscopy system and lean their heads back to reduce radiation exposure by increasing the distance. Wearing appropriate-fitting protective equipment is critical; otherwise, radiation will be absorbed by poorly protected areas of the body. The amount by which radiation exposure is diminished depends on multiple factors, including the magnitude of exposure and the fit of the protective equipment. In an audit of practice, Whittaker et al. found that most of the surgeons and staff were noncompliant with the use of a thyroid shield [39]. Thyroid collar protection with the standard 0.5 mm of lead has been shown to reduce the equivalent dose to the thyroid by a factor of 12, and the lighter 0.35 mm lead collars have been shown to provide a reduction in dose by a factor of 7 [40]. Because of discomfort from the collars, they were often worn loosely, leaving the upper portion of the thyroid exposed to scattered radiation. This action can lead to a false sense of security and injudicious fluoroscopy use that causes unnecessary absorption to the uncovered portion of the thyroid gland. Therefore, we believe that the proper wearing of protectors is important.

In addition, if the surgeon operates alone, it is effective to operate on the opposite side of the X-ray tube (the detection side). Furthermore, it is important to reduce the radiation exposure time to reduce the radiation exposure dose. The radiation time can be shortened by avoiding continuous radiation and using One Shot imaging. It was reported that intraoperative exposure can be reduced by using pulse X-ray radiation. Goodman et al. reported that radiation time could be reduced to 56.7% by using Pulse for X-ray radiation [41]. However, it is difficult for inexperienced residents to pay sufficient attention to X-ray radiation time. It was reported that the radiation time is significantly longer when a resident surgeon performs the procedure [42]. Therefore, senior surgeons will also need to provide guidance on radiation exposure. Carmichael et al. conducted a survey of 19 residents on their attitudes toward radiation exposure [43]. During the period under study, the expected number of radiation exposure by the 19 residents was 961, while the actual number of exposures was 2320. This was approximately 2.4 times the estimated amount. The survey, which further divided the residents into junior and senior residents, found that junior residents underestimated by more than 70%, while senior residents underestimated by less than 30%. Gendelberg et al. described the importance of instructing orthopedic residents on radiation exposure [44]. The course consists of a two-hour lecture on radiation safety and one-hour hands-on training. The average radiation exposure times for forearm fractures and distal radius fractures prior to the course were 41.2 and 28.1 s, respectively. In contrast, the average radiation exposure times after the course were 28.9 and 26.7 s, respectively. The training also had a lasting effect, and even one year after the training, the time of radiation exposure remained lower than before the training. These results indicate that attending a radiation safety program course can reduce the radiation exposure experienced by medical staff. We believe that radiation time is one of the factors that can be most easily reduced by medical staff awareness. Close communication is also important, not only with the residents but also with the radiologists, including specific instructions for each procedure.

This study also found that the scrub nurse beside the primary surgeon, who was next to the primary surgeon, was exposed to more radiation than the first assistant, who was closer to the fluoroscopic equipment. There are three types of ionizing radiation exposure sources: (1) direct exposure from the primary X-ray beam, (2) scattered radiation reflected from patient’s body or table, and (3) leakage from the X-ray tube [45]. There is no danger of being exposed to the primary X-ray beam unless the operator places his or her hand in the X-ray irradiation area during the procedure. Furthermore, because the amount of leaking X-rays is not large, it does not substantially affect the practitioner. The major radiation exposure risk for most medical staff, including pain physicians, originates from scatter radiation [42].

We think that the low exposure at the 80 cm and 100 cm heights of the first assistant is due to the radiation being absorbed after passing through the phantom. Yamashita et al. reported that direct radiation was attenuated to less than 100th after passing through the body [46]. Among the three principles of radiation exposure reduction that nurses can adopt, distance and isolation are the most important. We instruct our nurses to always wear protective eyewear, neck covers, and separate protectors during spinal surgery. It is also important to move away from the irradiation as much as possible [47]. Scattered radiation is reduced to 0.1% and 0.025% of primary radiation at distances of 3 feet (0.91 m) and 6 feet (1.82 m), respectively [48]. Alonso et al. reported that a protector is not necessary if the medical staff are 2 m away from the fluoroscopy device [49]. However, because it is difficult to open a distance of 2 m each time during irradiation, nurses at our hospital moved one step to the patient’s foot side during irradiation. Stepping away from the radiation device results in a 25–45% decrease in radiation exposure [31].

In the current study, anesthesiologists, circulation nurses, and radiology technicians were all in the low-dose range of 1 μGy/min or less. The estimated annual radiation exposure of the public not engaged in radiation was 1 mSv [50]. Even anesthesiology and circulation nurses are exposed to more radiation than the public. Therefore, the staff involved in and entering the operating room must be aware of radiation exposure. The limitations of this study are that only lateral fluoroscopy was used for validation, and measurements were not taken during the actual surgery.

## 5. Conclusions

We developed an aerial dose distribution map for lateral fluoroscopy of posterior spinal fusion. Radiation exposure was found to be the highest among primary surgeons. And it was found that the amount of exposure was higher for scrub nurses, who were located on the side of the primary surgeon, than for the first assistant, who was closer to the fluoroscopic equipment. Medical staff entering the operating room must be aware of radiation exposure.

## Data Availability

The data used to support the findings of this study are available from the corresponding author upon request.
